# Mannitol cannot reduce the mortality on acute severe traumatic brain injury (TBI) patients: a meta–analyses and systematic review

**DOI:** 10.1186/s41038-015-0006-8

**Published:** 2015-06-05

**Authors:** Kai Wang, Mingwei Sun, Hua Jiang, Xiao-ping Cao, Jun Zeng

**Affiliations:** 1 Department of Emergency Medicine, Affiliated Hospital of North Sichuan Medical College, Nanchong, 637000 P. R. China; 2 Department of Computational Mathematics and Biological Statistics, Metabolomics and Multidisciplinary Laboratory for Trauma Research, Institute for Emergency and Disaster Medicine, Sichuan Provincial People’s Hospital, Sichuan Academy of Medical Sciences, Chengdu, 610101 P. R. China; 3 Department of Acute Care Surgery, Sichuan Provincial People’s Hospital, Sichuan Academy of Medical Sciences, Chengdu, 610073 P. R. China

**Keywords:** Mannitol, TBI, Systematic review, Meta-analysis, Mortality, Intracranial pressure (ICP)

## Abstract

**Background:**

We aimed to systematically review the efficacy of mannitol (MTL) on patients with acute severe traumatic brain injury (TBI).

**Methods:**

Databases such as PubMed (US National Library of Medicine), CENTRAL (The Cochrane Library 2014, Issue 3), ISI (Web of Science: Science Citation Index Expanded), Chinese Biomedicine Database (CBM), and China Knowledge Resource Integrated Database (CNKI) have been searched for relevant studies published between 1 January 2003 and 1 October 2014. We have established inclusion and exclusion criteria to identify RCTs, which were suitable to be enrolled in the systematic review. The comparison group could be hypertonic saline (HS), hydroxyethyl starch, or others. The quality assessment was based on the Cochrane Handbook for Systematic Reviews of Interventions Version 5.0.1 and modified Jadad score scale. The major outcome was mortality, followed by the secondary outcomes such as neurological outcome, days on intensive care unit (ICU), and ventilator day. In addition, intracranial pressure (ICP), cerebral perfusion pressure (CPP), and mean arterial pressure (MAP) were used as the surrogate endpoints. Data synthesis and meta-analysis was conducted by using R (version 3.7-0.).

**Results:**

When 176 potential relevant literatures and abstracts have been screened, four RCTs met all the inclusion criteria and were enrolled for the meta-analysis. Amongst all the enrolled studies, two trials have provided the primary outcome data. There was no heterogeneity between two studies (*I*^2^ = 0 %) and a fixed model was used for meta-analysis (*n* = 53), pooled result indicated that the mortality was similar in mannitol intervention and control treatment, OR = 0.80, 95 % CI [0.27, 2.37], *P* = 0.38. We found that both mannitol and HS were efficient in decreasing the ICP. Furthermore, the effect of the HS on the ICP appeared to be more effective in the patients with diffuse brain injuries than mannitol did.

**Conclusions:**

As a conclusion, the mannitol therapy cannot reduce the mortality risk of acute severe traumatic brain injury. Current evidence does not support the mannitol as an effective treatment of acute severe traumatic brain injury. The well-designed randomized controlled trials are in urgent need to demonstrate the adoption of mannitol to acute severe traumatic brain injury.

## Background

Dehydration treatment is one of the main interventions that are adopted to prevent herniation of intracranial hypertension. Mannitol is widely used in China as a dehydrating agent in traumatic brain injury (TBI) patients with intracranial hypertension. Earlier studies indicated that dehydration therapy might decrease the intracranial pressure (ICP) of patients with TBI by reducing cerebral edema [1]. However, the excessive dosage of mannitol will penetrate from the blood into the brain, where it might cause the increase of the intracranial pressure (rebound phenomenon) and introduce the secondary brain cells damage accordingly [2, 3]. Therefore, the effectiveness of the treatment of mannitol for the intracranial hypertension in TBI is controversial [4, 5]. After the 1990s, a few new dehydration products have been introduced as the ICP reduction mediums, such as the hypertonic saline (HS) and the colloidal solution. Some clinical trials have shown that the hypertonic saline is more effective than mannitol in reducing the intracranial pressure and increase the cerebral perfusion pressure [5, 6]. However, there are studies with various results and conclusions [7, 8]. Single clinical trial usually has disadvantages by its sample size, design, or conduction. Clinical practitioners are puzzled by the chaos of the conflicted evidences and opinions to this end. The widespread use of mannitol is in great need of clarity optimal administration. There is uncertainty over the effectiveness of mannitol when compared to other ICP-lowering agents and other treatment without dehydrating agents. As a result, a systematic review is in urgent need, and this is why we present this study.

## Methods

### Strategy of data retrieving

Published literatures on the use of mannitol in severe traumatic brain injury patients were retrieved in the following databases: PubMed (US National Library of Medicine), The Cochrane Library (2014, Issue 3), ISI (Web of Science: Science Citation Index Expanded), Chinese Biomedicine Database (CBM), and China Knowledge Resource Integrated Database (CNKI). Letters have been sent to the first authors of the reports, asking them to assist in identifying any further trials that may have been conducted by them but not been reported publicly. Eligibility was determined by reading the reports of possible trials. The terms and strategies of retrieving are listed in the Table 1.

**Table 1:** Search terms and search strategy

Database	Searched items	Search strategy
PubMed	1. (Mannitol OR Mannit OR Mannite OR Osmosal OR Osmosteril OR Resectisol OR Aridol OR Bronchitol)	#1 AND #2 AND #3
	2. (“intracranial pressure” OR “intracranial hypotension” OR “intracranial hypertension” OR brain)	
	3.((randomized controlled trial[pt] OR controlled clinical trial[pt]OR (“Clinical Trials as Topic” [MeSH Major Topic])) NOT ((“Animals” [MeSH]) NOT (“Humans” [MeSH] AND “Animals” [MeSH]))	
ISI	1. Topic = (mannitol or Mannit or Mannite or Osmosal or Osmosteril or Resectisol or Aridol or Bronchitol)	1 and 2 and 3
	2. Topic = (intracranial pressure OR intracranial hypotension OR intracranial hypertension OR brain*)	
	3. TS = (clinical OR control* OR placebo OR random OR randomised OR randomized OR randomly OR random order OR random sequence OR random allocation OR randomly allocated OR at random)	
Cochrane	1.(mannitol OR Manit OR Mannite or Osmosal or Osmosteril or Resectisol or Aridol or Bronchitol)	1AND (2 OR 3 OR 4)
	2. “intracranial pressure”	
	3. “intracranial hypotension”	
	4. “intracranial hypertension”	
CBM	1. “mannitol” [Full field] 2. “mannitol” [MeSH])” 3. brain injury” [Full field] 4. “Head injury” [MeSH] 5. “Parietal damage” [Full field] 6. “Frontal damage” [Full field] 7. “Temporal damage” [Full field] 8. “Head injury” [Full field] 9. “Occipital damage” [Full field] 10. “Brain Injury” [MeSH] 11. “intracranial hypotension” [Full field] 12. “intracranial hypotension” [MeSH] 13. “Animal experiments” [Full field] 14. “Animal experiments” [MeSH]	(1 OR 2) AND (3 OR 4 OR 5 OR 6 OR 7 OR 8 OR 9 OR 10) AND (11 OR 12) NOT (13 OR 14)
CNKI	1. KY = mannitol 2. KY = brain injury 3. KY = intracranial hypotension 4. KY = animals	1 AND 2 AND 3 NOT 4

#### Inclusion criteria

Studies evaluating adult trauma patients with severe traumatic brain injuries and which included mannitol as an intervention were evaluated. The following data were required for inclusion: (1) study design: only RCTs set up with parallel control groups were selected, excluding self-control or crossover trials;(2) type of patients: adult trauma patients (age ≥18 years); (3) severe brain injures (Glasgow coma score <8) with cerebral edema. When indexes of the two groups such as genders, ages, pre-treatment ICP, osmolality levels, and Glasgow scores were matched, then the two groups were comparable; (4) intervention: the treatment group received mannitol in any dose for any duration, while the comparison group could be placebo controlled, different dose, different agent, or no agent; (5) reported one or more outcomes as following: (a) primary outcome: the mortality and (b) secondary outcome: (i) days on ICU and (ii) ventilator day; and (6) surrogate endpoints: ICP, cerebral perfusion pressure (CPP), and mean arterial pressure (MAP). The mortality is related with secondary and surrogate outcomes, so we collect them in the study.

#### Exclusion criteria

The following data were required for exclusion: (1) non-RCTs; (2) age ≤17 years, diagnosis of stroke, brain tumors, and non-traumatic brain injuries; (3) crossover studies; (4) did not address any primary or secondary outcomes as mentioned above; and (5) for those studies that did not describe the randomization methods, we attempted to contact the original authors. If the original authors did not provide a response or the randomization method proved inadequate, the articles were excluded.

#### Methodological quality evaluation

The methodological quality assessment table was based on the Cochrane Reviewers’ Handbook [9] and the modified Jadad scale [10, 11]. Data synthesis was conducted by R (R package version 3.7-0.) [12]. We followed the Preferred Reporting Items for Systematic Reviews and Meta-analyses (PRISMA) statement to report the research protocol, outcome, and relevant items in this systematic review [13].

### Limitations of the study

Though there are many published literatures regarding the use of mannitol in severe traumatic brain injury patients, few of them have met the high quality standards of evidence-based medicine. There is insufficient reliable evidence to make suggestions on the administration of mannitol and many unanswered questions on the optimal use of mannitol in severe traumatic brain injury patients.

## Results and discussion

### Study identification and selection

**Fig. 1:**
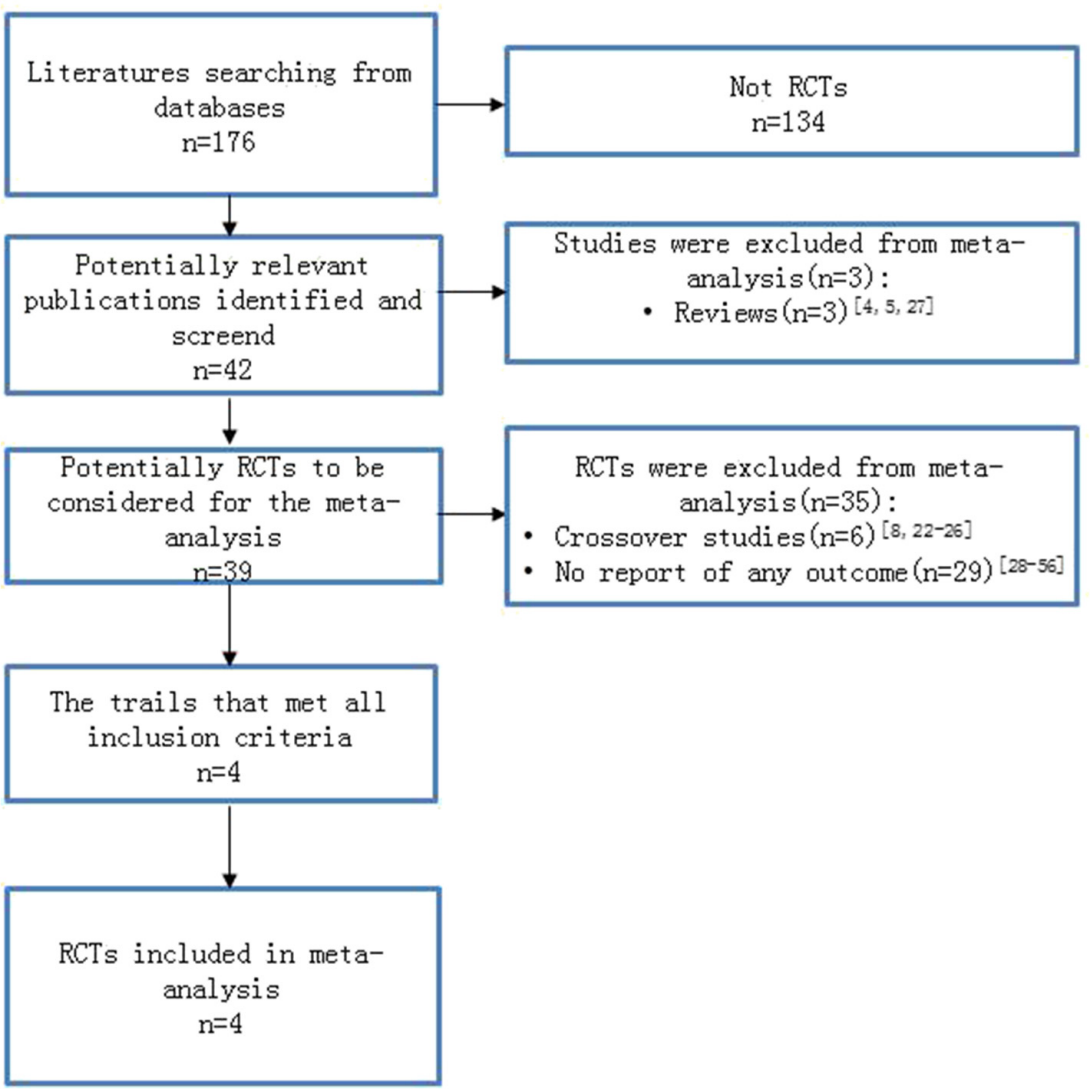
Literature searching and selection

There were in total 176 potentially relevant titles, abstracts, and articles that have been screened. Initial screening resulted in 42 candidate studies [4, 5, 8, 14–52]. Figure 1 shows the details of the selection process and the reasons for exclusion. There were four trails that met all inclusion criteria and were included in the final meta-analysis. The characteristics of these included trials are listed in Tables 2 and 3.

**Table 2:** Methodological characteristics of studies

Author	Number of patients	Randomization	Concealment	Blinded	Withdraw	Single or multicenter	Balance of two groups	Total modified Jadad score
Vialet et al. [49]	20	Randomization table	Envelopes concealment	Single Blinded	0	Single center	Yes	7/7
Harutjunyan et al. [50]	32	Computer randomly	Not reported	Not reported	0	Single center	Yes	3/7
Francony et al. [51]	17	Randomization table	Envelopes concealment	Single blinded	0	Multi-center	Yes	7/7
Cottenceau et al. [52]	56	Based on blocks of four	Envelopes concealment	Double blinded	0	Single center	Yes	7/7

**Table 3:** The definition of secondary outcomes and surrogated endpoints of included trials

Author	Patients	Interventions	Outcomes	Definition of surrogated endpoints
Vialet et al. [49]	Severe head injury (GCS <8); follow up to 3 months	The mannitol group receives 20 % mannitol solution. The hypertonic saline group received 7.5 % hypertonic saline. The infused volume was the same for the both solutions: 2 mL/kg body weight in 20 min	Death and neurological disability reported	HS caused a greater decrease in ICP than mannitol
Harutjunyan et al. [50]	Severe neuronal damage (GCS <8).	Seventeen patients received 7.2 % NaCl/HES 200/0.5 and 15 received mannitol	Death reported	HES caused a greater decrease in ICP than mannitol (57 vs. 48 %; *p* <0.01)
Francony et al. [51]	Severe brain injury (trauma, stroke); they were aged >18 years and had sustained elevated ICP of >20 mmHg for >10 min. Follow up to 3 months	The mannitol group received 231 mL of 20 % mannitol. The hypertonic saline group received 100 mL of 7.45 % hypertonic saline. Both to be administrated via the central venous catheter in 20 min	ICP, CPP, MAP reported	ICP decreasing did not differ between the two groups. Mannitol if effective than HS in CPP
Cottenceau et al. [52]	Severe head injury (GCS <8); follow up to 6 months	The mannitol group received 4 mL/Kg of 20 % mannitol. The hypertonic saline group received 2 mL/Kg of 7.5 % hypertonic saline. Both be administrated in 20 min	Neurological outcome reported	Neurological outcome and ICP decreasing did not differ between the groups

### Major outcome: mortality

**Fig. 2:**
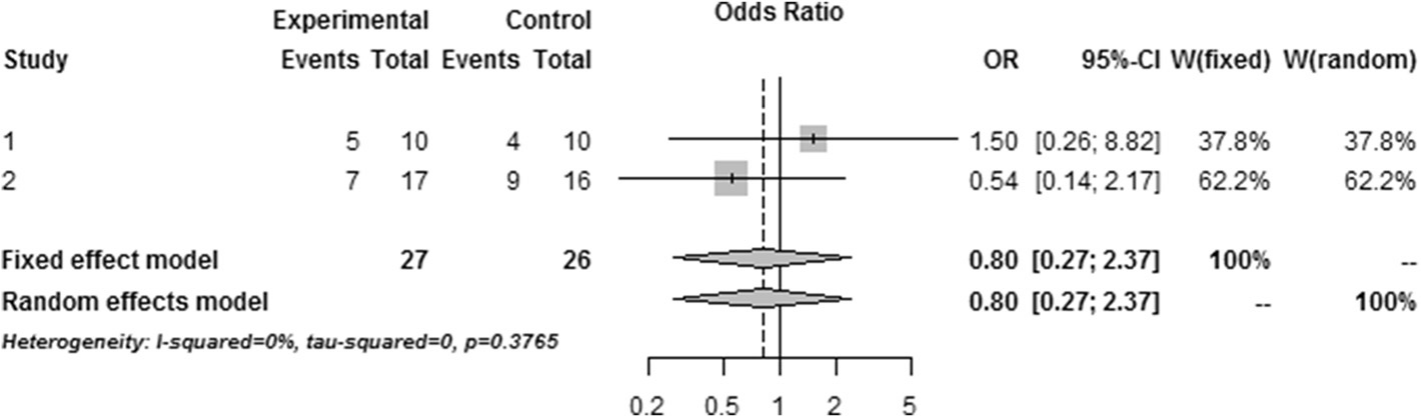
Comparison 1: mannitol versus hypertonic saline. Outcome 1 mortality

Amongst all the enrolled studies, Vialet et al. [49] and Harutjunyan et al. [50] have provided the primary outcome (mortality) (Table 4, Fig. 2). There is no heterogeneity between two studies (*I*^2^ = 0 %), and a fixed model was used for the meta-analysis (*n* = 53), pooled result indicated that the mortality was similar in mannitol intervention and control treatment, OR = 0.80, 95 % CI [0, 27, 2.37], *P* = 0.38.

**Table 4:** Mortality of included studies

Studies	Mannitol group	Control group	*P* value
Vialet et al. [49]	5/10 (50 %)	4/10 (40 %)	*P* >0.05
Harutjunyan et al. [50]	7/17 (41.2 %)	9/16 (56.2 %)	*P* >0.05

### Secondary outcomes

In terms of the report of the secondary outcomes and surrogate endpoints, four RCTs were not uniformed and the data synthesis was not available. Hence, we have conducted a qualitative analysis afterward. There was no difference between two groups in neurological outcome (Glasgow outcome scale (GOS) 5/10 vs. 6/10; *P* >0.05) and days on ICU (23.3 ± 14.8 vs. 22.8 ± 15.5; *P* >0.05). No trial reported ventilator day.

### Surrogated endpoints

#### ICP

Mannitol therapy may have a detrimental effect on decrease ICP when compared to hypertonic saline therapy. Vialet et al. [49] compared 20 % mannitol group with 7.5 % HS group. The result suggested that the mean number (13.3 ± 14.6 vs. 6.9 ± 5.6 episodes) of intracranial hypertension episodes (ICP >25 mmHg) per day and the daily duration (131 ± 123 vs. 67 ± 85 min) of intracranial hypertension episodes were significantly higher in the mannitol group (*P* <0.01) than those in the HS group [49]. The rate of clinical failure was also significantly higher in the mannitol group (7 of 10 vs. 1 of 10 patients; *P* <0.01) than that in the HS group [49]. Harutjunyan et al. [50] compared 15 % mannitol to 7.2 % hypertonic saline hydroxyethyl starch 200/0.5 (7.2 % NaCl/HES 200/0.5). NaCl/HES 200/0.5 (7.2 %) caused a greater decrease in the ICP than mannitol did (57 vs. 48 %; *P* <0.01) [50]. Both groups decreased the ICP to below 15 mmHg; but the mean time was significantly longer in the mannitol group (8.7 vs. 6 min; *P* <0.01) than that in the HS group. Francony et al. [51] and Cottenceau et al. [52] have shown that the spectrum of the ICP decrease was significantly larger in the HS group than in the mannitol group, and the spectrum was about 10 % over the entire stage. The changes of ICP across the four studies are summarized in Table 5.

**Table 5:** ICP of included studies

		Measurement of surrogate index			Measurement of surrogate index	
		Number of episodes per day			Total duration of episodes, min/day	
Vialet et al. [49]	Intervention: 20 % M 2 mL/Kg	13.3 ± 14.2^**^				95 ± 92^**^
	Control: 7.5 % HSS 2 mL/Kg	6.8 ± 5.5^**^				62 ± 81^**^
		T0	T30	T60	T90	T120
Harutjunyan et al. [50]	Intervention: 15 % M 1.4 mL/Kg	23 [19–30]	12 [6–19]^*^	14 [7–20]^*,**^		
	Control: 7.2 % NaCl/HES 1.8 mL/Kg	22 [19–31]	10 [6–14]^*^	11 [5–18]		
Cottenceau et al. [52]	Intervention: 20 % M 4 mL/Kg	16.3 ± 9.3	10.5 ± 6.8^*^			13.6 ± 7.5^*^
	Control: 7.5 % HSS 2 mL/Kg	17.9 ± 9.9	13.9 ± 7.8^*^			13.9 ± 7.8^*^
		Δ%	Δ%	Δ%	Δ%	Δ%
Francony et al. [51]	Intervention: 20 % M 231 mL	31 ± 6	−41 ± 23^*^	−45 ± 19^*^	−35 ± 12^*^	−32 ± 12^*^
	Control: 7.45 % HSS 100 mL	27 ± 3	−37 ± 18^*^	−35 ± 14^*^	−31 ± 15^*^	−23 ± 10^*^

*P <0.05 vs. T0; **P < 0.05 vs. mannitol

#### CPP

Mannitol therapy may have a small detrimental effect in increasing CCP when compared to hypertonic saline therapy. Mannitol therapy may only have a beneficial effect in improving the blood supply of local brain tissue. Vialet et al. [49] has indicated that there was no significant difference in the mean number (3.1 ± 3.6 vs. 4.0 ± 4.6 episodes) of cerebral perfusion hypotension episodes (CPP <70 mmHg) per day and the daily duration (62 ± 107 vs. 52 ± 83 min) of cerebral perfusion hypotension episodes between the two groups (*P* >0.05) [49]. Harutjunyan et al. [50] has shown that the CPP had significantly sharp increase in both groups after the start of the infusion (*P* <0.0001), and the HS group was significantly higher than the mannitol group at 30 min after the beginning of the infusion (*P* <0.05) [50]. The maximum increase occurred at 30 min after the beginning of the infusion in both groups, and the HS group increased more than the mannitol group did (27 vs. 18 %; *P* <0.05) [50]. Francony et al. [51] suggested that mannitol could only effectively improve the CPP and the blood supply of the local brain tissue [51]. Cottenceau et al. [52] suggested that the maximum of CPP increase occurred at 30 min after the beginning of the infusion in both groups, but there was no significantly difference between the 20 % mannitol and the 7.5 % HS group [52]. The changes of CCP across the four studies are summarized in Table 6.

**Table 6:** CPP of included studies

		Measurement of surrogate index			Measurement of surrogate index	
		Number of episodes per day			Total duration of episodes, min/day	
Vialet et al. [49]	Intervention: 20 % M 2 mL/Kg	3.1 ± 3.6^**^			62 ± 107^**^	
	Control: 7.5 % HSS 2 mL/Kg	4.0 ± 4.6^**^			58 ± 83^**^	
		T0	T30	T60	T90	T120
Harutjunyan et al. [50]	Intervention: 15 % M 1.4 mL/Kg	61 [47–71]	72 [60–93]^*^	73 [58–88]^*,**^		
	Control: 7.2 % NaCl/HES 1.8 mL/Kg	60 [39–78]	75 [62–86]^*,**^	69 [56–89]^*^		
Cottenceau et al. [52]	Intervention: 20 % M 4 mL/Kg	72.1 ± 12.8	76.9 ± 17.4^8^			74.7 ± 13.3^*^
	Control: 7.5 % HSS 2 mL/Kg	72.8 ± 15.1	79.3 ± 11.6^*^			74.3 ± 13.1^*^
		Δ%	Δ%	Δ%	Δ%	Δ%
Francony et al. [51]	Intervention: 20 % M 231 mL	75 ± 15	+21 ± 23^*^	+22 ± 21^*^	+14 ± 15^*^	+17 ± 14^*^
	Control: 7.45 % HSS 100 mL	81 ± 12	+21 ± 23^*^	+9 ± 10^*^	+8 ± 11^*^	+7 ± 6^*^

*P <0.05 vs. T0; **P <0.05 vs. mannitol

#### MAP

Mannitol therapy may have no beneficial effect in increasing MAP when compared to hypertonic saline therapy. Harutjunyan et al. [50] shown that MAP had a significantly sharp increase in 15 % mannitol and 7.2 % NaCl/HES 200/0.5 after the beginning of the infusion (*P* <0.05). There was no significant difference in the increase of MAP between two groups (5.8 vs. 7.6 %), but the maximum increase occurred significantly shorter in the mannitol group than in the HS group (10 vs. 30 min) [50]. Francony et al. [51] and Cottenceau et al. [52] have shown in their research that there was no significant difference in MAP between the 20 % mannitol and the 7.5 % HS. The changes of MAP across the four studies are summarized in Table 7.

**Table 7:** MAP of included studies

		T0	T30	T60	T90	T120
Harutjunyan et al. [50]	Intervention: 15 % M 1.4 mL/Kg	84 [68–92]	81 [69–106]	82 [68–108]		
	Control: 7.2 % NaCl/HES 1.8 mL/Kg	84 [64–98]	85 [74–100]^*,**^	84 [63–94]^*^		
Cottenceau et al. [52]	Intervention: 20 %M 4 mL/Kg	87.6 ± 12.2	87.4 ± 11.6			87.9 ± 10.9
	Control: 7.5 % HSS 2 mL/Kg	90.6 ± 12.6	91.2 ± 10			87.2 ± 10.5
		Δ%	Δ%	Δ%	Δ%	Δ%
Francony et al. [51]	Intervention: 20 % M 231 mL	106 ± 16	+2 ± 9	+1 ± 10	0 ± 10^*^	+2 ± 7
	Control: 7.45 %Hss 100 mL	108 ± 13	−5 ± 7	−2 ± 7	−3 ± 7^*^	−1 ± 5

*P <0.05 vs. T0; **P <0.05 vs. mannitol

#### Plasma osmolality, urine, and serum sodium

Mannitol therapy may have no difference in elevating the plasma osmolality and serum sodium when compared to hypertonic saline therapy. Mannitol therapy may have a small beneficial effect in increasing the urine output. But the single trial was too small for reliable conclusion. Vialet et al. [49] suggested that the plasma osmolality and serum sodium elevated significantly in both the 20 % mannitol group and the 7.5 % HS group after the beginning of the infusion (*P* <0.05), but there was no significant difference between the two groups. Harutjunyan et al. [50] showed the serum sodium increased in both the 15 % mannitol and the 7.2 % NaCl/HES 200/0.5 (*P* <0.05), although there was no significant difference between the two groups. Francony et al. suggested that mannitol caused a significantly greater increase in urine output (*P* 0.05) than HS [51]. Cottenceau et al. suggested that the HS caused a significantly greater increase of serum sodium (*P* = 0.0000), although mannitol caused a significantly decrease of it (*P* = 0.0000) [52]. The changes of plasma osmolality and sodium across the four studies are summarized in Table 8.

**Table 8:** Plasma osmolality and serum sodium of included studies

	Measurement of surrogate index		Intervention	Control
Vialet et al. [49]	Serum sodium (Δ%)		+1.3 ± 10.1^*^	+4.7 ± 8.2^*^
Francony et al. [51]	Serum sodium (Δ%)		−1.7 ± 3.2^**^	+2.1 ± 1.4^**^
	Urine output (mL/h)		306 ± 174^**^	114 ± 72^**^
Harutjunyan et al. [50]	Serum sodium	T0		143 (136–148)
		T30		148 (144–153)^*^
	Plasma	T0	286 (270–315)^*^	284 (273–300)^*^
	Osmolality	T30	295 (278–327)^*^	300 (284–319)^*^
Cottenceau et al. [52]	Serum sodium	T0	141.3 ± 5.1	144.2 ± 5.1
		T30	139.1 ± 4.1^*,**^	148.3 ± 5.2^*,**^

*P <0.05 vs. T0; **P <0.05 vs. mannitol

## Conclusions

Published literatures regarding the use of mannitol in severe traumatic brain injury patients have rarely met the high quality standards of evidence-based medicine. This systematic review has revealed that the mannitol therapy cannot reduce death risk for TBI patients suffering from raised ICP.

Four RCTs retrieved by this study which have found reduced intracranial pressure (ICP): two RCTs suggested that hypertonic saline was superior to mannitol, and the time of maximized effect was earlier than that of mannitol; whilst the other two RCTs have proposed mannitol was equally on the impact of ICP comparing with hypertonic saline. In the elevation of CPP and MAP, one RCT found that hypertonic saline was superior to mannitol, whilst the other three RCTs considered no significant difference between the two agents. One RCT showed that mannitol might have a beneficial effect on cerebral hemodynamic when compare to hypertonic saline. However, some domestic studies have shown that the 23.4 % HS have faster and longer effect than the 20 % mannitol did in the treatment of raised ICP of severe brain injury [53–56]. Thus, the points of those researchers on this issue are varied and contradicted with each other.

Currently, mannitol is widely used as a part of conventional therapy of the TBI patients who suffer from intracranial pressure hypertension. Based on the most credible evidence from this study, mannitol therapy cannot reduce the mortality risk in this type of patients. Well-designed randomized controlled trials are urgently needed to demonstrate the efficacy of mannitol to acute severe traumatic brain injury.

## Abbreviations

CI: confidence interval
CPP: cerebral perfusion pressure
GCS: Glasgow coma score
HES: hydroxyethyl starch
HS: hypertonic saline
ICP: intracranial pressure
ICU: intensive unit care
MAP: mean arterial pressure
OR: odds ratio
PRISMA: Preferred Reporting Items for Systematic Reviews and Meta-Analyses
RCT: randomized controlled trial
TBI: traumatic brain injury
